# Double-twist cylinders in liquid crystalline cholesteric blue phases observed by transmission electron microscopy

**DOI:** 10.1038/srep16180

**Published:** 2015-11-04

**Authors:** Shu Tanaka, Hiroyuki Yoshida, Yuto Kawata, Ryusuke Kuwahara, Ryuji Nishi, Masanori Ozaki

**Affiliations:** 1 Division of Electrical, Electronic and Information Engineering, Osaka University, 2-1 Yamada-oka, Suita, Osaka 565-0871, Japan; 2 Research Center for Ultra-High Voltage Electron Microscopy, Osaka University, 7-1 Mihogaoka, Ibaraki, Osaka, 567-0047, Japan

## Abstract

Cholesteric blue phases are liquid crystalline phases in which the constituent rod-like molecules spontaneously form three-dimensional, helical structures. Despite theoretical predictions that they are composed of cylindrical substructures within which the liquid crystal molecules are doubly twisted, real space observation of the arrangement of such structures had not been performed. Through transmission electron microscopy of photopolymerized blue phases with controlled lattice plane orientations, we report real space observation and comparison of the lattice structures of blue phases I and II. The two systems show distinctly different contrasts, reflecting the theoretically predicted, body centred and simple cubic arrangement of the double-twist cylinders. Transmission electron microscopy also reveals different tendencies of the two blue phases to align on unidirectionally rubbed surfaces. We thus show that TEM observation of alignment-controlled, photopolymerized liquid crystals can be a powerful tool to investigate complex liquid crystalline order.

The orientation of rod-like liquid crystalline molecules is described by a unit vector referred to as the director^1^. In materials with chiral constituents, the director self-organizes into helical structures with one or three-dimensional periodicities, known as the cholesteric and cholesteric blue phases (BPs), respectively^1^,^2^. BPs typically appear in materials with strong chiralities such that the periodicity is less than 500 nm and, depending on their structure, are further classified into three subphases, BPs I, II, and III^3^. BPs I and II possess body-centered and simple cubic structures, while BP III is amorphous with short-ranged three-dimensional ordering^4^. The three-dimensional structure confers properties such as optical Bragg reflection, macroscopic optical isotropy, and sub-millisecond electro-optic response, making them potentially useful for displays, photonic switches, and lasers^5^,^6^,^7^,^8^. Furthermore, the last decade has witnessed enormous development in materials, with BPs being combined with polymers, nanoparticles, and molecular dimers to provide superior stability^9^,^10^,^11^, and improvements being made in material properties such as the dielectric anisotropy to reduce the driving voltage^12^,^13^. BPs are thus viewed as next-generation electro-optic materials that can beat the slow (~ms) response of nematic liquid crystals used in current displays^7^.

Despite advances in materials and application proposals, real-space verification of the three-dimensional structure of BPs remains a challenge. The current understanding of the BP structure is based on a theoretical model which has been shown to minimize the free-energy associated with director distortions^2^,^14^,^15^. In this model, the liquid crystal molecules form a cylindrical substructure within which the director is parallel to the cylinder axis at the centre, and twists by 45° along all directions perpendicular to itself^14^. To fill three-dimensional space, the double-twist cylinders (DTCs) stack spontaneously; a simple cubic lattice with space group O^2^ (P4_2_32) is formed when the DTCs stack such that the cylinder axes point in the three orthogonal directions and touch each other, and a body-centred cubic lattice with space group O^8^ (I4_1_32) results when every second DTC is removed from the simple cubic lattice in each direction. From diffraction experiments using light with various energies, BP I has been assigned the O^8^ symmetry and BP II, the O^2^ symmetry^15^,^16^,^17^. However, although the symmetry of BPs is generally accepted, there are only a limited number of reports of observing the BP lattice structure in real space. Previous observation attempts by transmission electron microscopy (TEM) and atomic force microscopy (AFM) combined with freeze-fracture^18^ or thermal quenching^19^,^20^ techniques had succeeded in visualizing bi-periodic structures distinct from other phases. However, no compelling evidence had been presented as to the distribution of the DTCs, mainly owing to the fact that the BP lattice orientation was not controlled in the specimens under investigation. Moreover, only BPs I and III have been observed^21^, with BP II remaining yet to be observed. A more recent study reporting the lattice structure of polymer-stabilized BP I by means of confocal laser scanning microscopy (CLSM) has also reported periodic modulations in the reflected light intensity^22^; however, the image formation mechanism is not completely understood, and the observed patterns do not necessarily match the DTC distribution in the lattice.

Here, real-space observation and comparison of the DTC distributions in BPs I and II are reported through TEM observation of a photopolymerizable BP. Photopolymerizable liquid crystals behave in a manner similar to conventional, low-molecular-weight liquid crystals, but can be quenched *in situ* upon UV irradiation, thereby enabling the preparation of high-quality films with well-controlled lattice orientations. Here, the two BPs are observed along the same direction ([100] direction) to directly compare the contrast in each phase. The two BPs exhibit distinctly different textures, each agreeing with the DTC arrangement predicted by theory. Moreover, wide-field observation of the films reveals a clear difference in the tendencies of the two phases to form a monodomain on unidirectionally rubbed substrates – a characteristic believed to be attributed to the difference in the lattice structure, or more precisely, the arrangement of the topological defect lines. We thus show that TEM observation of alignment-controlled, photopolymerized liquid crystals can be a powerful tool to investigate complex liquid crystalline order.

## Results

### Preparation and optical characterization of quenched BPs

Conventional low molecular-weight liquid crystal molecules cannot be observed by TEM because of the high-vacuum environment required. Here, we prepare polymer films with quenched BP order using a photopolymerizable liquid crystal doped with a strongly twisting chiral dopant^23^. The sample exhibits thermotropic BPs I and II before polymerization, but upon triggering photopolymerization via UV irradiation, the molecular order is quenched and phase transitions are no longer observed. To prepare films with controlled BP lattice orientations, the liquid crystal was infiltrated in a sandwich cell composed of two glass substrates coated with a unidirectionally rubbed, polyimide-based alignment layer. The substrates impose molecular alignment parallel to the substrate, and orients the [100] direction normal to the substrates as the sample is heated from the cholesteric phase (for BP I) or cooled from the isotropic phase (for BP II). The sample was heated and cooled repetitively within the corresponding BP temperature ranges to obtain the [100] orientation across the whole sample, and then irradiated by a strong UV light source to quench the molecular orientation.

**Figure 1:**
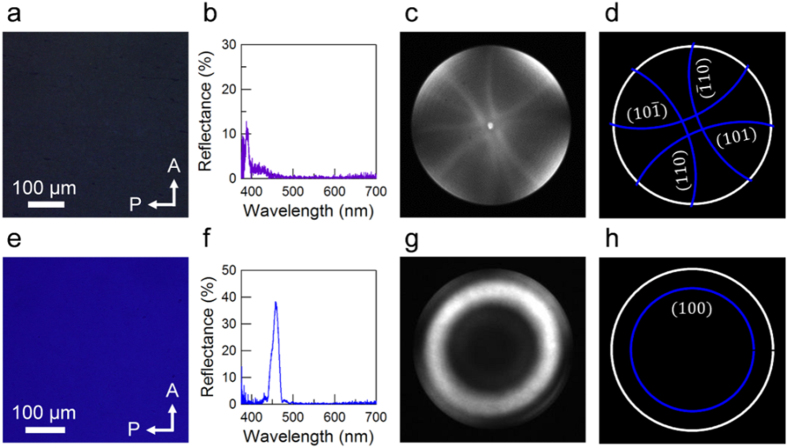
Optical characterization of the quenched BPs. (**a–d**) POM image (**a**), reflection spectrum (**b**), Kossel diagram (**c**) and its simulation (**d**) of the quenched BP I film. (**e–h**) POM image (**e**), reflection spectrum (**f**), Kossel diagram (**g**), and its simulation (**h**) of the quenched BP II film.

The sample orientation and lattice constants were confirmed prior to TEM observation from polarized optical microscopy (POM) and microspectroscopy. Figure 1 shows the POM images, reflection spectra, and Kossel diagrams^24^ of the quenched BPs I and II samples. A strong reflection peak appeared at approximately 387 nm and 455 nm, corresponding to the colors observed by POM. Considering the selection rule for Bragg reflection associated with the structure of each BP, the reflection peaks are attributed to the (200) and (100) planes for BPs I and II, respectively. Assuming an average refractive index of 1.64 and 1.61 for the two wavelengths (measured by spectroscopic ellipsometry, Supplementary Fig. S1), the lattice constants are evaluated as 236 nm and 141 nm. The Kossel diagram is a diffraction pattern for a converging monochromatic light incident on the BP crystal and provides information on its periodicity and symmetry. Using refractive index values of 1.63 and 1.62 for BPs I and II for the wavelength at which the Kossel diagram was observed (420 nm), the experimental Kossel diagrams are reproduced successfully when lattice constants of 245 nm and 143 nm are assumed. The lattice constant turns out to be slightly larger in the film-plane direction than in the film-normal direction, possibly because of the anisotropy in the polymerization-induced shrinking of the film^23^. Nevertheless, periodic molecular orientation is confirmed in the photopolymerized BP films.

### TEM observation of the orientation-controlled BPs I and II

**Figure 2:**
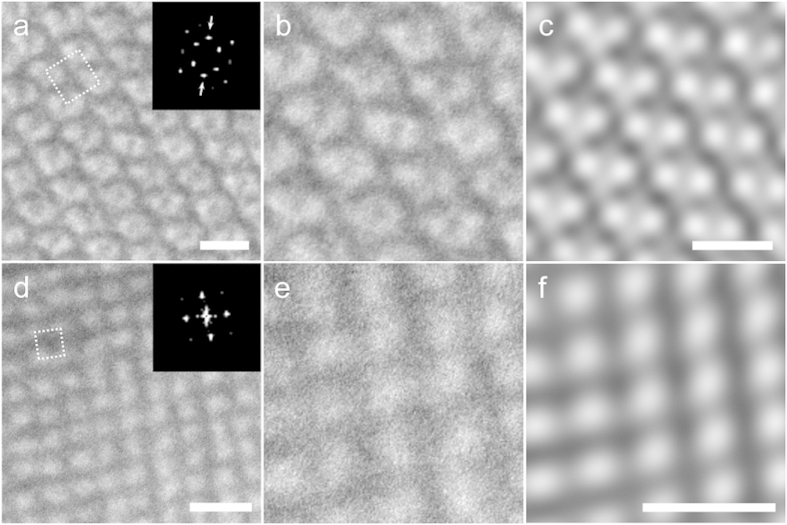
Typical TEM images of quenched BP I and BP II. (**a**) TEM image and corresponding FFT pattern of BP I. (**b**) A close-up view of the central region in (**a**). (**c**) Inverse FFT (IFFT) image of (**a**), after filtering out the non-periodic components of FFT patterns shown in (**a**). (**d**) TEM image and corresponding FFT pattern of BP II. (**e**) A close-up view of the central region in (**d**). (**f**) Corresponding IFFT image of (**d**), filtering out non-periodic components of FFT patterns shown in (**d**). Scale bars, 300 nm.

TEM observation was performed on ultramicrotomed sections of BPs I and II films with a thickness of approximately 80 nm. Cuts were made parallel to the film plane, resulting in thin-films with the (100) plane oriented parallel to the film. Figure 2a,d show typical TEM images obtained for the BPs I and II films. One can immediately identify a four-fold symmetry in the observed contrasts, which agrees with the expected symmetry when the BP lattice is observed from the [100] direction. Fast Fourier transform (FFT) analysis of the images yields a strong set of peaks with nearly four-fold symmetry for both BPs; the slight deviation of the peaks from a perfect four-fold symmetry is attributed to the anisotropic compression of the polymerized film along the microtome cut (Supplementary Fig. S2). The periodicity of the contrast estimated by measuring the periodicities at various locations are 238 ± 10 nm for BP I and 136 ± 5 nm for BP II (average ± standard deviation, N = 50). These values correspond approximately to the periodicities of peaks giving the strongest intensities in the FFT pattern; see insets of Fig. 2a,d. A periodic contrast with symmetry and periodicity agreeing with that obtained from optical measurements is thus observed in the TEM. A closer inspection of the TEM images (Fig. 2b,e) reveals a difference between the two BPs. In the BP I lattice, there exists a two-fold axis along the diagonal of the square unit cell (as marked with arrows in Fig. 2a, with periodicity 168 ± 8 nm, N = 40), whereas the BP II unit cell only has four-fold symmetry. This difference is observed in the FFT of the TEM image in that a set of peaks of two-fold symmetry is observed only in BP I. The reconstructed image after filtering out the non-periodic components (Fig. 2c,f) emphasizes this difference in contrast.

**Figure 3:**
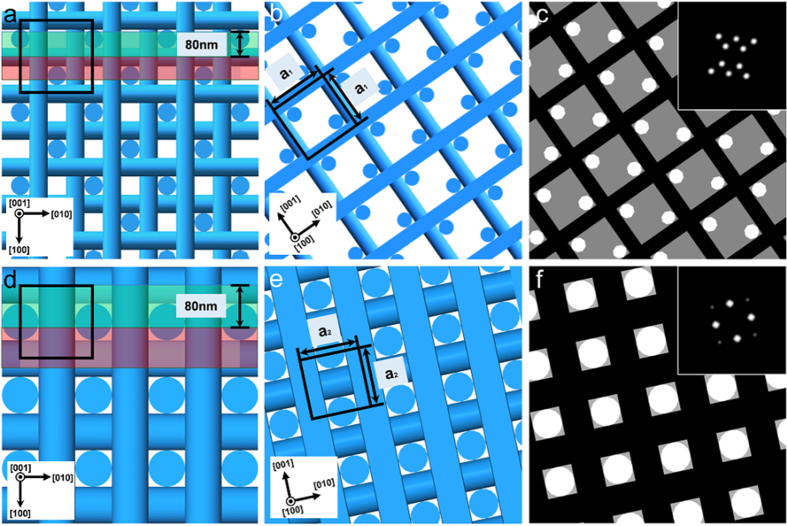
Structure model of BPs and cross sectional structures. (**a**) Theoretically predicted arrangement of the DTCs in BP I viewed along the [001] direction. (**b**) Ultra-thin section of BP I corresponding to the region shaded green in (**a**), viewed along the [100] direction. (**c**) Same as (**b**) but with after changing the contrast of the DTCs depending on the orientation. Inset shows the FFT image. (**d**) Theoretically predicted arrangement of the DTCs in BP II viewed along the [001] direction. (**e**) Ultra-thin section of BP II corresponding to the region shaded green in (**d**), viewed along the [100] direction. (**f**) Same as (**e**) but with after changing the contrast of the DTCs depending on the orientation. Inset shows the FFT image.

We argue that the observed textures are direct reflections of the DTC distributions in the two BPs. Previous studies on quenched liquid crystal films have suggested several factors affecting the contrast appearing in the TEM, including difference in the mass-thickness density^25^, cut-induced surface topography^20^, anisotropic etching caused by electron radiation^26^, and diffraction contrast^27^. While the contribution of each effect to the overall contrast has not been clarified, previous studies all agree that the TEM image appears bright where the liquid crystal director is oriented perpendicular to the plane of the slice, and dark where the director is parallel. By observing the contrast of a long-pitch cholesteric liquid crystal (ChLC) prepared by adjusting the amount of chiral dopant, we have confirmed that our material system is consistent with the previous studies (Supplementary Fig. S3). Considering that thickness modulations on the order of 10 nm were observed on the microtomed ChLC film, we suspect the cut-induced thickness modulation to be one of the more dominant causes in creating the image contrast. Based on the above, and considering that the director is on average oriented parallel to the cylinder axis within a DTC, the DTCs should appear bright where they are oriented perpendicular to the slice plane and dark where it is parallel. Fig. 3a,d show the theoretically proposed arrangement of the DTCs viewed along the [001] direction. Because of the small thickness (~80 nm) of the TEM slice compared to the lattice constant (a_1_ = 236 nm and a_2_ = 141 nm for BPs I and II), the number of DTCs contained in a single slice is less than the number of DTCs existing in a unit cell. If we assume that the thin sections shaded in green in Fig. 3a,d were produced by the ultramicrotoming process, structures shown in Fig. 3b,e are obtained. Changing the contrast of the figure so that the DTCs oriented perpendicular and parallel to the section appear bright and dark, respectively, and the region without the DTCs has intermediate brightness, one obtains textures shown in Fig. 3c,f, which are strikingly similar to those obtained in experiment. The FFT patterns also agree with experiment, with BP I possessing two sets of strong peaks with four-fold symmetry and one set of peaks with two-fold symmetry, and BP II only possessing two sets of peaks with four-fold symmetry. One may argue that the disordered regions (disclinations) that exist between the DTCs is another plausible cause for the contrast, because such regions have reduced molecular order^28^ and thus can be considered to have lower densities than the bulk^29^. However, this is ruled out since, if the disclinations were the cause of the contrast, the observed periodicity would be different from what is observed in experiment (Supplementary Fig. S4).

**Figure 4:**
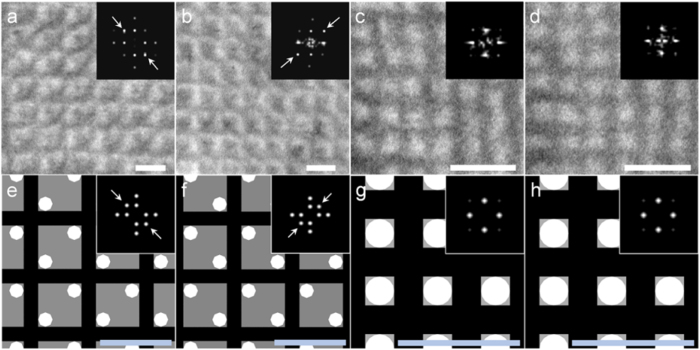
TEM images of serial sections of quenched BP I and BP II. (**a,b**) TEM images and corresponding FFT patterns of serial sections of BP I. (**c,d**) TEM images and corresponding FFT patterns of serial sections of BP II. (**e,f**) DTC distribution within a thin section of BP I after adjusting contrast as in Fig. 3c. Sections correspond to those shaded in red (**e**) and green (**f**) in Fig. 3a. (**g**,**h**) DTC distribution within a thin section of BP II after adjusting contrast as in Fig. 3f. Sections correspond to those shaded in red (**g**) and green (**h**) in Fig. 3d. Scale bars, 300 nm.

Our argument is further supported from the observation of serial sections. Consecutive slices were collected on a single-slit grid mesh coated with formvar and observed at the same relative positions by using the knife mark as a guide. Figure 4a–d show the TEM images for two consecutive slices in BPs I and II, respectively. Although the contrast is reduced due to the additional formvar layer on the TEM grid, one can recognize that for BP I, the contrast changes between consecutive slices, with the two-fold axis appearing to be rotated by 90° (see FFT images in inset). On the other hand, no obvious change is observed for BP II. This can be explained by considering the small thickness of the microtomed section compared to the BP lattice constant. Assuming the first slice to correspond to the section shaded red in Fig. 3 and the second slice to the section shaded green, for BP I, the relative positions of the DTCs oriented perpendicular to the film changes with respect to those oriented parallel to the film (Fig. 3e,f). As the FFT images indicate, this causes the axis of two-fold symmetry to be rotated by 90°. In contrast, the arrangement of the DTCs, and hence FFT patterns remain constant in BP II, because of the higher symmetry and smaller lattice constant.

### Observation of BP I and BP II domains grown on uniaxially rubbed substrates

**Figure 5:**
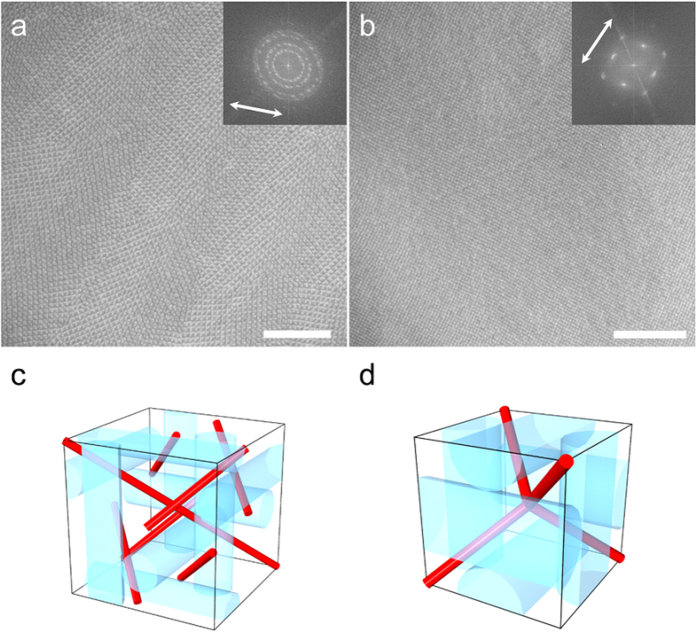
Wide-field TEM images and schematic illustration of disclination lines BPs I and II. (**a**) Wide-field TEM image of quenched BP I and corresponding FFT pattern. (**b**) Wide-field TEM image of quenched BP II and corresponding FFT pattern. Arrows indicate the direction of orientational rubbing. Scale bars, 3 μm. (**c**) Unit cell of BP I with the 7 independent disclination lines shown in red. (**d**) Unit cell of BP II with the disclination network shown in red.

Figure 5a,b show TEM images of BPs I and II at a slightly lower magnification than from the previous figures. Despite being grown on substrates with the same alignment conditions, the two samples show a clear difference in the domain size. In BP I, the sample is polydomain, with the azimuthal orientation of the unit cells changing on the order of several μm. In contrast, in BPII, no domain boundaries are observed and the sample appears completely monodomain. We have observed other locations within the film and found BP II to be free of domain boundaries (Supplementary Fig. S5). This difference is also apparent in the FFT patterns of the two samples (inset of Fig. 5a,b), as the FFT peaks form a band that is distributed quasi-isotropically in BP I, whereas they show a clear four-fold symmetry in BP II (the deformation in the FFT pattern is again due to the cut-induced compression by the microtome process; see Supplementary Fig. S6). The domain size of BP I measured at 58 domains had a log-normal distribution with a mode of 6.7 μm^2^ and logarithmic standard deviation of 0.97, implying the growth rate to be size-independent. Quantitative measurement of the azimuthal orientation at 98 domains provide further support for the weak tendency of BP I to orient along the rubbing direction (Supplementary Fig. S7). This is in stark contrast to BP II, which is seen to align one of its < 100 > axes along the rubbing direction. The behaviour is also counterintuitive, as BP I, which is grown from the lower temperature cholesteric phase with uniform alignment, is polydomain, and BP II, which is grown from the isotropic liquid with no preferred orientation, is monodomain.

An important question to consider is where the difference in the alignment behaviour originates from. Because both phases are grown on substrates with the same anchoring conditions, we speculate that the phenomenon is attributed to the difference in the structure, or more precisely, the difference in the configuration of the disclination lines in the two BPs. Figure 5c,d illustrate the disclination lines proposed to be present in the BPs unit cells^15^. In a BP I unit cell, there are 7 disclination lines running through the unit cell without intersecting with each other, while in BP II, the disclinations are connected and form a network with a diamond-like structure. The disclinations are topologically protected and elastic in nature, and so domain boundaries are formed at an energy cost of bending, terminating, or creating a disclination. Because the free energy of a BP containing domain boundaries is higher than that of a perfect crystal, there is an elastic torque generated to reduce the mismatch in the crystal orientation. However, in BP I where the disclinations are separated, there are many local minima in the free energy, because mending of one disclination requires a different disclination to be deformed, and thus costs additional energy. In contrast, for BP II in which only a single disclination exists as a network, the mismatch in azimuthal orientation can be reduced simply by shortening the disclination length. The only minimum in the free energy is the global minimum, in which all unit cells have the same orientation, i.e., a monodomain is obtained. The difference in the tendencies of the two BPs to form a monodomain on rubbed substrates have been pointed out by Kizel’ and Prokhorov, who investigated the optical activity of BPs^16^. Our results not only provide real space confirmation of their results, but also quantitative information on the distribution of domain size and azimuthal orientation. Such information, combined with theoretical simulations, may in the future help clarify the orientation mechanism of BPs and its relation to material parameters.

## Discussion

TEM observation of quenched BP films with controlled lattice orientations allows visualization of the DTC distribution in BPs, thereby providing direct evidence of the structure predicted by the Landau-de Gennes theory. The symmetry and periodicity of the observed structures not only agree two-dimensionally, but agrees three-dimensionally, as shown from the observation of serial sections. While there are several studies that have discussed the structure of BPs by performing TEM observation on thermally quenched or freeze-fractured samples^18^,^19^,^20^, previous studies had not succeeded in providing compelling evidence for the DTC distribution as in our study. This study is also the first report of observing the BP II lattice, which is more temperature-sensitive than BP I and therefore difficult to quench by rapid cooling. Our results are mainly due to the following two advantages of photopolymerizable liquid crystals: (i) the orientation of the liquid crystal can be controlled easily prior to photopolymerization through conventional alignment layers developed for low-molecular weight liquid crystals, and (ii) photopolymerization-induced quenching can be performed *in situ* without moving the sample (for example, dipping into a liquid propane bath^18^), thereby greatly improving the quality of alignment within the resultant film.

We also comment on the possibility of applying our strategy to investigate other complex liquid crystal-based structures. It is known that an electric field induces a change in BP crystal structure from cubic to orthorhombic, tetragonal, or hexagonal, depending on the dielectric anisotropy and direction of field^30^,^31^. Moreover, recent theoretical studies have demonstrated the existence of even more complex structures in BPs under strong confinement^32^,^33^ or under the influence of electric fields^31^,^34^. Chiral liquid crystals thus form structures that are difficult if not impossible to fabricate artificially, with potential for switchable optics and photonics applications^35^. To realize these structures, specific conditions must be met, such as strong surface anchoring, small thickness, accurate temperature control, and application of an electric field; however, as the samples used in our technique can be prepared in a sandwich cell structure, such conditions can be met easily. We therefore anticipate that our approach would be a powerful tool to study liquid crystal structures yet unobserved.

## Methods

### Materials

A photopolymerizable BP was prepared by mixing a reactive mesogen (Merck, RMM-141C) and a strongly twisting chiral dopant (Merck, CD-X) at a weight ratio of 95.5:4.5. The phase sequence of the material before polymerization was determined from polarized optical microscopy while heating and cooling the sample at a rate of 0.1 °C/min (performed with a commercial hotstage, Linkam, LTS-350). The observed phase sequence was cholesteric (Ch) (54.4 °C)/BP I (55.5 °C)/BP II (58.3 °C)/isotropic phase (Iso) for heating, and Ch (51.0 °C)/BP I (55.2 °C)/BP II (58.1 °C)/Iso for the cooling process.

### Fabrication of BP film and optical measurements

The films with quenched BPs I and II order were fabricated using sandwich cells assembled from glass substrates coated with polyimide (JSR, AL1254) and rubbed unidirectionally. The rubbing direction was aligned to the edge of the rectangular-shaped substrates and the sandwich cells were assembled by hand; we estimate the rubbing direction to have an accuracy better than ± 3 degrees. To achieve the desired orientation, BP I was heated to 54.8 °C from the cholesteric phase, while BP II was cooled to 57.2 °C from the isotropic phase. For BP I, a temperature cycling process was performed to achieve uniform orientation throughout the sample. The sample was heated from 54.8 °C to 55.0 °C at a rate of 0.1 °C/min and held for 10 minutes, and then cooled from 55.0 °C to 54.8 °C at a rate of 0.1 °C/min and held for 10 minutes; this process was repeated for 8 hours. BP II, on the other hand, showed uniform alignment without the heating process. After confirming uniform orientation in the whole field of view, the samples were irradiated with UV light of intensity approximately 400 mW/cm^2^ (measured at 365 nm) for 120 sec. to induce polymerization. The sample temperature was kept at 54.8 °C for BP I and 57.2 °C for BP II. After polymerization, the upper substrate was removed, and small portions (~1 × 1 mm) of the films were cut. Measurement of optical spectra were performed with a spectrometer (Ocean Optics, USB4000, fibre-diameter 600 μm) coupled to a POM equipped with a × 10 objective. Kossel patterns were observed at λ = 420 nm using a × 100 objective with a N.A. of 0.90. Refractive index measured by spectroscopic ellipsometry (J.A. Woollam, M-2000) were used to evaluate the lattice constants.

### TEM observation and data analysis

After optical measurements, the BP films were dehydrated with ethanol and embedded in epoxy resin (Quetol 812 set, NisshinEM, 340). The resin was cut along the sheet plane with an ultramicrotome (LEICA, EM UC7) to an approximate thickness of 80 nm. The microtomed sections were collected on 150 μm mesh grids, and observed by a TEM (Hitachi, H-7500) with an accelerating voltage of 80 kV. For the serial sections, a slit grid with a supporting formvar layer was used. Analyses of the TEM images were performed using a commercial data analysis software (Wave Metrics, Igor Pro). The periodicities of the contrast were measured at various locations where the azimuthal orientation of the BP lattice was nearly parallel to the knife mark. Because of the compression along the knife mark, the periodicity appeared shorter along the direction of the cut: the periodicities were 229 nm (N = 25) and 131 nm (N = 25) parallel to the knife mark, and 247 nm (N = 25) and 140 nm (N = 25) perpendicular to the knife mark for BPs I and II, respectively. The values written text are the average along the two directions. In the discussion of the alignment behaviour, the rubbing direction was estimated by acquiring low magnification images such that the whole film could be observed, and then measuring its orientation.

## Additional Information

**How to cite this article**: Tanaka, S. *et al.* Double-twist cylinders in liquid crystalline cholesteric blue phases observed by transmission electron microscopy. *Sci. Rep.* **5**, 16180; doi: 10.1038/srep16180 (2015).

## Supplementary Material

Supplementary Information
