# Supplementary material for: Double-twist cylinders in liquid crystalline cholesteric blue phases observed by transmission electron microscopy
Source: Sci Rep. 2015 Nov 4;5:16180. doi: 10.1038/srep16180 (PMC4632023; doi:10.1038/srep16180)
Supplement: Supplementary Information [file srep16180-s1.pdf]

Supplementary Information for

"Double-twist cylinders in liquid crystalline cholesteric blue phases observed by transmission electron microscopy"

S. Tanaka[1], H. Yoshida[1], Y. Kawata[1], R. Kuwahara[2], R. Nishi[2], and M. Ozaki[1]

Affiliations:

[1]Division of Electrical, Electronic and Information Engineering, Graduate School of Engineerng, Osaka University, 2-1 Yamadaoka, Suita, Osaka 565-0871, Japan

[2]Research Center for Ultra-High Voltage Electron Microscopy Osaka University, 7-1 Mihogaoka, Ibaraki, Osaka, 567-0047, Japan

Contents:

7 Supplementary Figures

1. Refractive index dispersion of quenched BP I and BP II sample.

The refractive indices of the photopolymerizable BP films were evaluated by means of spectroscopic ellipsometry (J.A. Woollam, M-2000). Because the films have no absorption resonances in the visible region, Cauchy model was employed to describe the wavelength dispersion.

Figure S1 shows the wavelength dependence of the refractive index for BPs I and II. At 387 nm and 455 nm where Bragg reflection is observed for BPs I and II, respectively, the corresponding refractive indices are 1.64 and 1.61. The refractive indices for λ = 420 nm, at which the Kossel diagrams were observed, are 1.63 and 1.62.

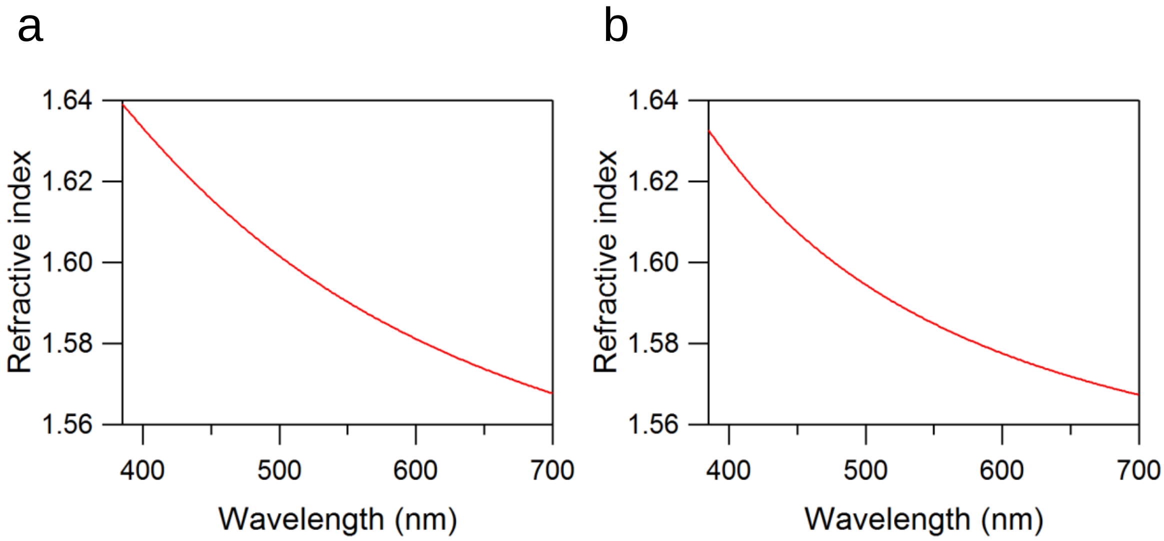

Fig. S1 Dispersion of the refractive index for BPs I and II films. (**a**) Wavelength dependence of the refractive index for BP I. (**b**) Wavelength dependence of the refractive index for BP II.

2. Microtoming-induced compression in a photopolymerized liquid crystal.

Compression of the specimen along the cut direction is an artifact often encountered in soft materials[1]. Figure S2 shows a TEM image of BP I where a knife mark is visible in the field of view. The knife edge is sharp, but not perfectly sharp, thus causing deformation close to the surface of the sample section. Knife marks are artificial furrows caused by places where the knife edge is damaged. The image is compressed in the direction of the knife mark, thereby supporting that distortion of the image is caused by the microtoming process.

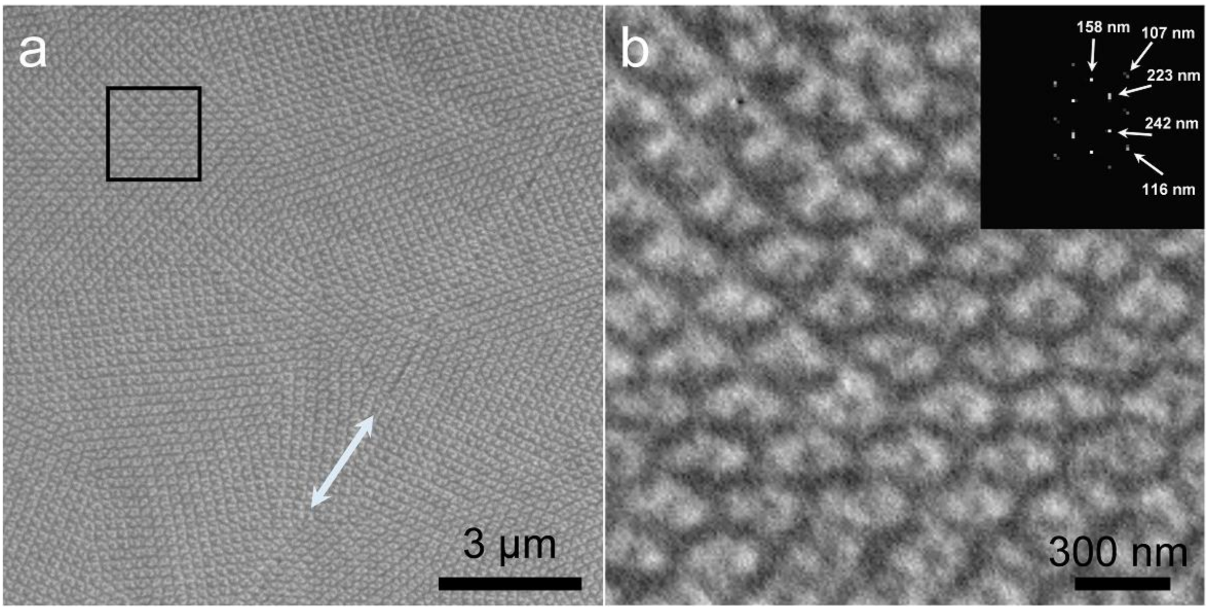

Fig. S2: Microtoming-induced compression in a quenched BP I. (**a**) TEM image of BP I where a knife mark is visible in the field of view. Arrow indicates the cut direction. (**b**) A close up view of the black boxed area in (**a**) and corresponding FFT pattern.

3. TEM contrast in a long-pitch cholesteric liquid crystal film.

A long-pitch cholesteric liquid crystal was prepared by adjusting the concentration of the chiral dopant to 0.2 wt%. The cholesteric pitch was first determined optically by the Grandjean-Cano-method[2], using a glass sandwich cell with a wedge-angle of 0.31 °. As seen in the POM image of Fig. S3a, a discontinuous change in the number of half-pitches along the wedge direction causes a periodic array of disclination lines to be observed. The pitch of the cholesteric helix, p, can be evaluated from the spacing of the disclination line, Λ, and the wedge angle, θ, according to the equation $\tan\theta = p/2\Lambda$. The periodicity of the disclination lines is 1070 μm (average measured at 10 points), which yields a pitch length of approximately 12 μm.

After photopolymerizing the sample, its cross-section was observed in the TEM. The sample was embedded in epoxy resin following the procedure described in text, but the cut direction was adjusted for cross-sectional observation. Figure S3b shows a typical TEM image, where the dark strip corresponds to the cross-section of the cholesteric liquid crystal. The black arrow indicates the helical axis, and the liquid crystal director is oriented parallel to the section at the interface with the epoxy. Periodic bands of dark and bright regions are observed with a periodicity of 6.3 μm, approximately corresponding to the half-pitch of the sample. The brightness in the TEM therefore varies with the director orientation within the specimen. Comparing the modulation in brightness with the cholesteric twist, one sees that the regions where the liquid crystal director is parallel to the section plane appear dark, whereas the regions with perpendicular director orientation appear bright.

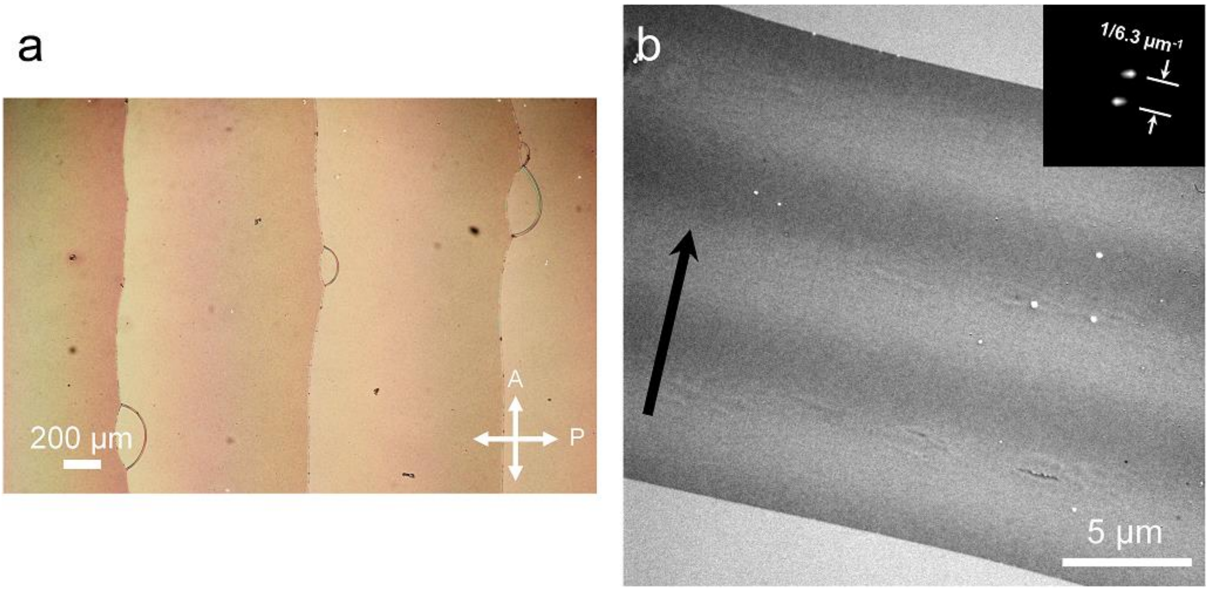

Fig. S3 TEM contrast observed in a long-pitch cholesteric liquid crystal film. (**a**) POM image of the long-pitch cholesteric liquid crystal. In a wedge-shaped cell, discontinuous change of the pitch induces periodic disclination lines. (**b**) Cross-sectional TEM image of the long-pitch cholesteric liquid crystal. The black arrow indicates the direction of the helical axis, and the liquid crystal director is oriented in the slice plane at the edges of the film.

4. Cross-sectional structures of disclinations

The unit cell of BPs I and II not only contain DTCs, but also regions of disorder where the orientation of the LC director cannot be defined unanimously (disclinations)[3]. Considering that such regions typically have reduced molecular order[4] and thus can be considered to have lower densities than the bulk[5], disclinations may also affect the image contrast. We show that this possibility is ruled out by showing that the observed contrast of disclinations would be different from that observed in experiment if the disclinations were the source of the image contrast. Figure S4a and c show cross sectional structures of disclinations shaded in green in Fig. 3a and d of the manuscript. Figure S4b and d are obtained by changing the contrast so that disclinations and the region without the disclinations appear bright and dark, respectively. These textures are clearly dissimilar to the observed structures, and the periodicities as observed from the FFT patterns also do not agree with experiment. The observed contrast therefore cannot be explained by the distribution of the disclinations.

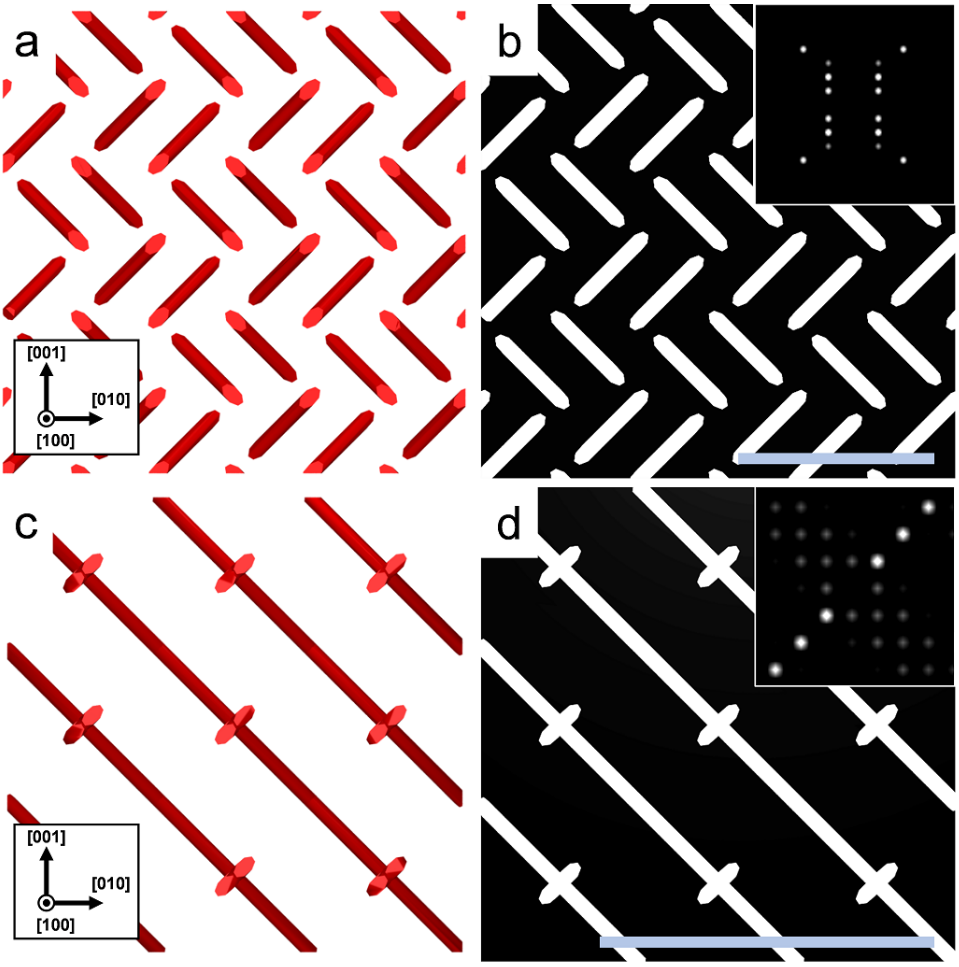

Fig. S4 Cross sectional structures of disclinations of BPs I and BP II. (**a**) Ultra-thin section of disclinations of BP I corresponding to the region shaded green in Fig. 3a, viewed along the [100] direction. 3 × 3 unit cells are shown. (**b**) Same as (**a**) but with after changing the contrast of the disclinations depending on the degree of molecular (**c**) Ultra-thin section of disclinations of BP II corresponding to the region shaded green in Fig. 3d, viewed along the [100] direction. 3 × 3 unit cells are shown. (**d**) Same as (**c**) but with after changing the contrast of the disclinations depending on the degree of molecular. Inset shows the FFT image. Scale bars, 300 nm.

5. Wide-field TEM observation of BPs I and II

TEM observations were performed at different locations from those in Fig. 5 of the manuscript. Figure S5 shows TEM images of BPs I and II at two different locations, clearly representing the polydomain nature of BP I, and monodomain nature of BP II.

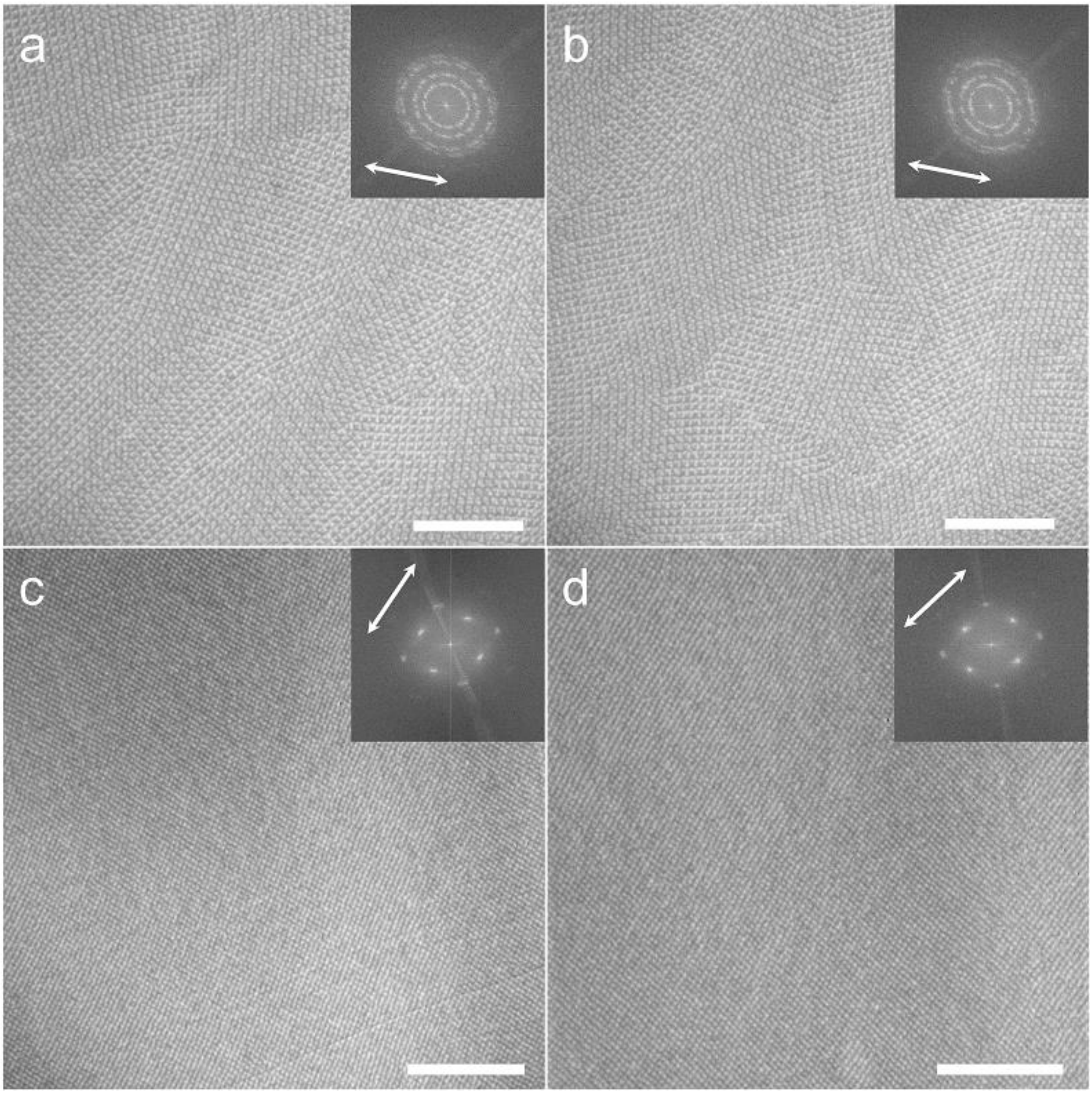

Fig. S5 Wide-field TEM image of quenched BP I and quenched BP II. (**a**, **b**) TEM image of BP I at two different locations within the same section as that shown in Fig. 5a. (**c**) TEM image of BP II at a different location within the same section as that shown in Fig. 5b, and (**d**) TEM image of BP II in another section. Arrows indicate the direction of orientational rubbing. Scale bars 3 μm.

Figure S6 shows a TEM image of BP I with the knife mark in the field of view (arrow in image). The FFT pattern of the image shows a quasi-isotropically distributed band, because

of the polydomain nature of BP I; however, the band is clearly deformed along the direction of cut. An elongation in the FFT pattern along the cut direction indicates that the film has been compressed.

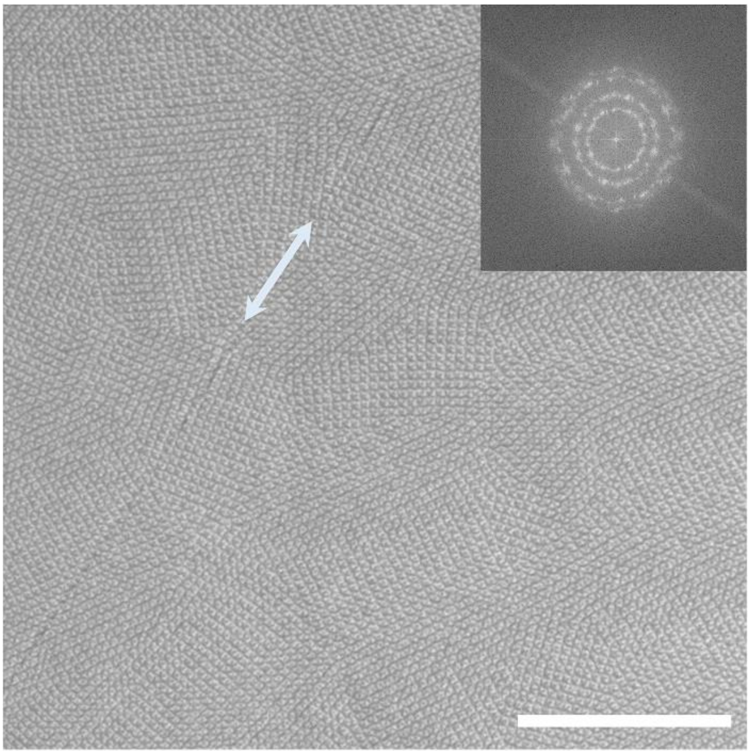

Fig. S6 Compression of the BP lattice along the cut-direction. Low magnification TEM image of BP I acquired so that the knife-mark would be in the field-of view. The FFT image in the inset shows that the lattice is compressed in the direction of the knife-mark. Arrow indicates a cutting knife direction trace. Scale bar 5 μm.

Figure S7 shows the histograms of the domain size and azimuthal orientation angle for BP I. As described in the main text, the domain size was measured at 58 domains and the orientation angle was measured at 98 domains. The domain size had a log-normal distribution with a mode of 6.7 μm^2 and logarithmic standard deviation of 0.97. The azimuthal orientation of the lattice indicated only a weak tendency to orient along the rubbing direction, agreeing with the quasi-isotropic FFT pattern observed.

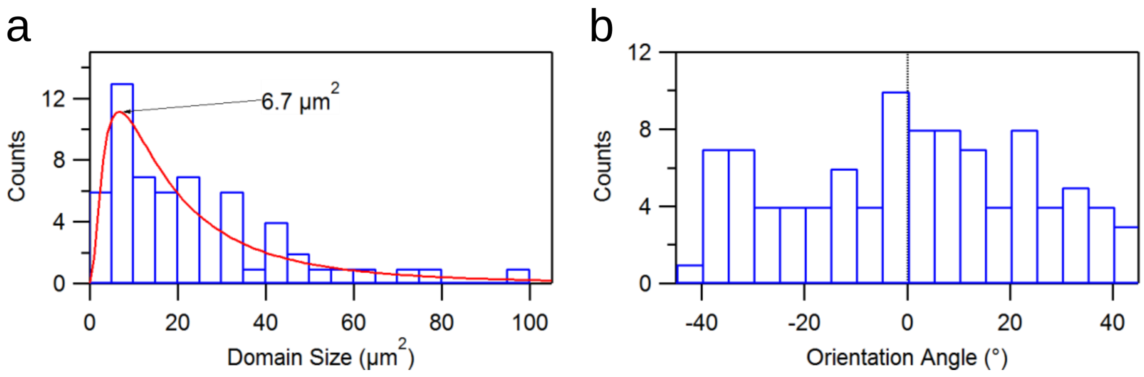

Fig. S7: The distribution of domain size and azimuthal orientation of quenched BP I. (**a**) Histogram of the domain size for BP I measured at 58 domains. (**b**) Histogram of the angle difference between the rubbing direction and the azimuthal orientation for BP I measured at 98 domains.

Supplementary References:

1. Sawyer, L.C. & Grubb, D. T. *Polymer Microscopy* (Chapman & Hall, London, 1996).
2. Cano, R. *Bull. Soc. Fr. Mineral. Cristallogr.* **91**, 20–27 (1968).
3. Meiboom, S., Sethna, J. P, Anderson, P. W. & Brinkman, W. F. Theory of the Blue Phase of Cholesteric Liquid Crystals. *Phys. Rev. Lett.* **46**, 1216-1219 (1981).
4. Zgura, I., Moldovan, R., Beica, T. & Frunza, S. Temperature dependence of the density of some liquid crystals in the alkyl cyanobiphenyl series. *Cryst. Res. Technol.* **44**, 883-888 (2009).
5. Schopohl, N. & Sluckin, T. J. Defect Core Structure in Nematic Liquid Crystals. *Phys. Rev. Lett.* **59**, 2582-2584 (1987).
